# The genome sequence of the Pacific oyster, *Magallana gigas *(Thunberg, 1793)

**DOI:** 10.12688/wellcomeopenres.22255.2

**Published:** 2025-02-03

**Authors:** Rob Mrowicki, Rebekka Uhl, Marine Biological Association Genome Acquisition Lab, Darwin Tree of Life Barcoding collective, Wellcome Sanger Institute Tree of Life Management, Samples and Laboratory team, Wellcome Sanger Institute Scientific Operations: Sequencing Operations, Wellcome Sanger Institute Tree of Life Core Informatics team, Tree of Life Core Informatics collective, Darwin Tree of Life Consortium

**Affiliations:** 1 The Marine Biological Association, Plymouth, England, UK

**Keywords:** Magallana gigas, Pacific oyster, genome sequence, chromosomal, Ostreida

## Abstract

We present a genome assembly from an individual *Magallana gigas* (the Pacific oyster; Mollusca; Bivalvia; Ostreida; Ostreidae). The genome sequence is 564.0 megabases in span. Most of the assembly is scaffolded into 10 chromosomal pseudomolecules. The mitochondrial genome has also been assembled and is 18.23 kilobases in length. Gene annotation of this assembly on Ensembl identified 19,775 protein-coding genes.

## Species taxonomy

Eukaryota; Opisthokonta; Metazoa; Eumetazoa; Bilateria; Protostomia; Spiralia; Lophotrochozoa; Mollusca; Bivalvia; Autobranchia; Pteriomorphia; Ostreida; Ostreoidea; Ostreidae; *Magallana*; *Magallana gigas* (Thunberg, 1793) (NCBI:txid2171618).

## Background

The Pacific oyster *Magallana gigas*, formerly known as *Crassostrea gigas,* is an invasive species now commonly found across Europe. Its native range is in Northeast and Southeast Asia. *M gigas* has a deeply cupped left valve and a flat right valve, both with corresponding deep ridges along its margins ( Hughes, 2008). It settles on hard surfaces and can form reef structures, where it feeds by filter feeding at high filtration rates ( Troost, 2010).

The oyster was first introduced to Europe after a decline in the European oyster ( *Ostrea edulis*) due to disease and severe winters. Since its introduction in 1964, it has been introduced in 66 countries, with wild populations found in 17 of these ( Wood *et al.*, 2021). Many studies have investigated competition between the Pacific and the European oysters, with some suggesting that the similar feeding preferences between the two oysters make them highly competitive, although others suggest that their co-habitation can allow niche partitioning within different tidal zones ( Ezgeta-Balić *et al.*, 2020; Zwerschke *et al.*, 2018). This may be further explored by comparing the now-published genomes of both species ( Adkins *et al.*, 2023).

*M. gigas* has the highest annual production of all aquaculture organisms in the world, credited to its plasticity in highly exploited environments full of anthropogenic stressors ( Hedgecock *et al.*, 2005; Tran *et al.*, 2022). With new genomic data becoming available, studies have used the chromosome-level genome of *M. gigas* to examine its plasticity in response to environmental stressors and disease ( Li *et al.*, 2021; Qi *et al.*, 2021; Yao *et al.*, 2022). A complete genome sequence will thereby provide further information to elucidate these functions, which will be beneficial in understanding its role in aquaculture and its threat to wild populations.

## Genome sequence report

**Figure 1.**
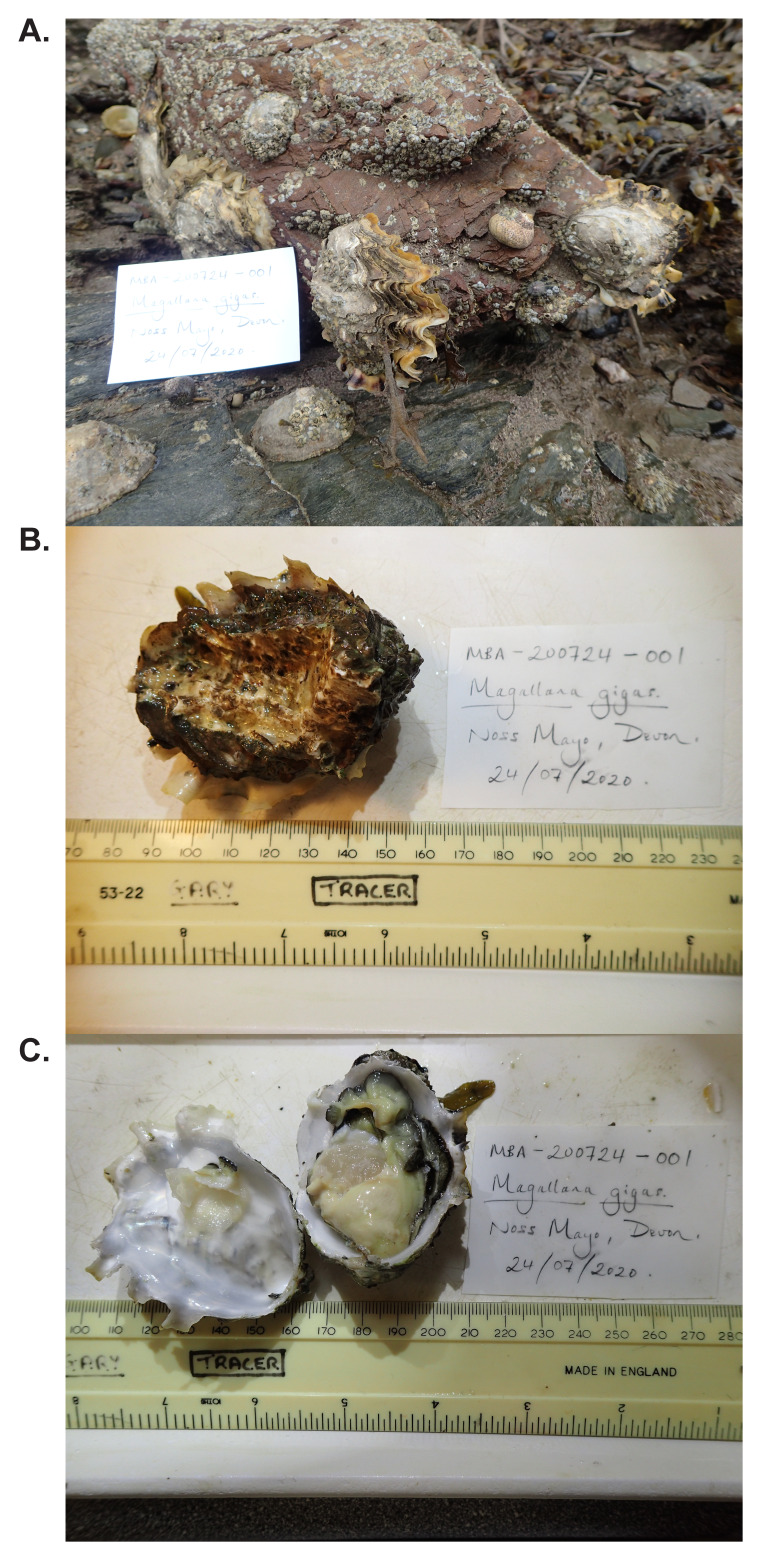
Photographs of the *Magallana gigas* (xbMagGiga1) specimen used for genome sequencing. **A** The whole animal attached to the substrate where it was found. **B**. The animal after being removed from the substrate. **C**. Internal view of both valves with tissue still attached after dissection.

The genome was sequenced from a specimen of *Magallana gigas* ( Figure 1) collected from Noss Mayo, Devon, UK (50.31, -4.05). A total of 48-fold coverage in Pacific Biosciences single-molecule HiFi long reads was generated. Primary assembly contigs were scaffolded with chromosome conformation Hi-C data. Manual assembly curation corrected 108 missing joins or mis-joins and removed 68 haplotypic duplications, reducing the assembly length by 13.48% and the scaffold number by 55.22%, and decreasing the scaffold N50 by 5.73%.

**Figure 2.**
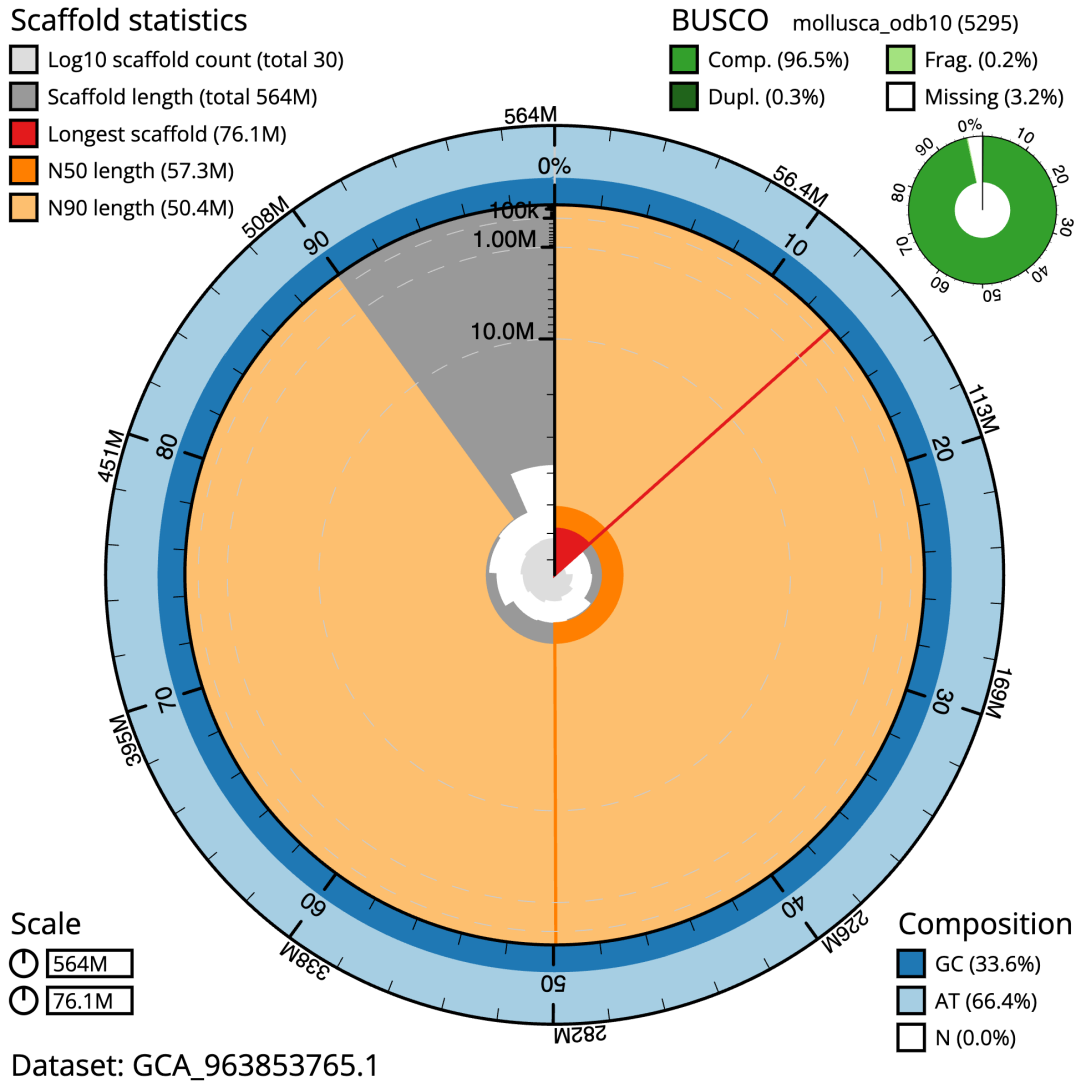
Genome assembly of *Magallana gigas*, xbMagGiga1.1: metrics. The BlobToolKit snail plot shows N50 metrics and BUSCO gene completeness. The main plot is divided into 1,000 size-ordered bins around the circumference with each bin representing 0.1% of the 564,004,028 bp assembly. The distribution of scaffold lengths is shown in dark grey with the plot radius scaled to the longest scaffold present in the assembly (76,070,991 bp, shown in red). Orange and pale-orange arcs show the N50 and N90 scaffold lengths (57,274,926 and 50,364,239 bp), respectively. The pale grey spiral shows the cumulative scaffold count on a log scale with white scale lines showing successive orders of magnitude. The blue and pale-blue area around the outside of the plot shows the distribution of GC, AT and N percentages in the same bins as the inner plot. A summary of complete, fragmented, duplicated and missing BUSCO genes in the mollusca_odb10 set is shown in the top right. An interactive version of this figure is available at https://blobtoolkit.genomehubs.org/view/Magallana_gigas/dataset/GCA_963853765.1/snail.

**Figure 3.**
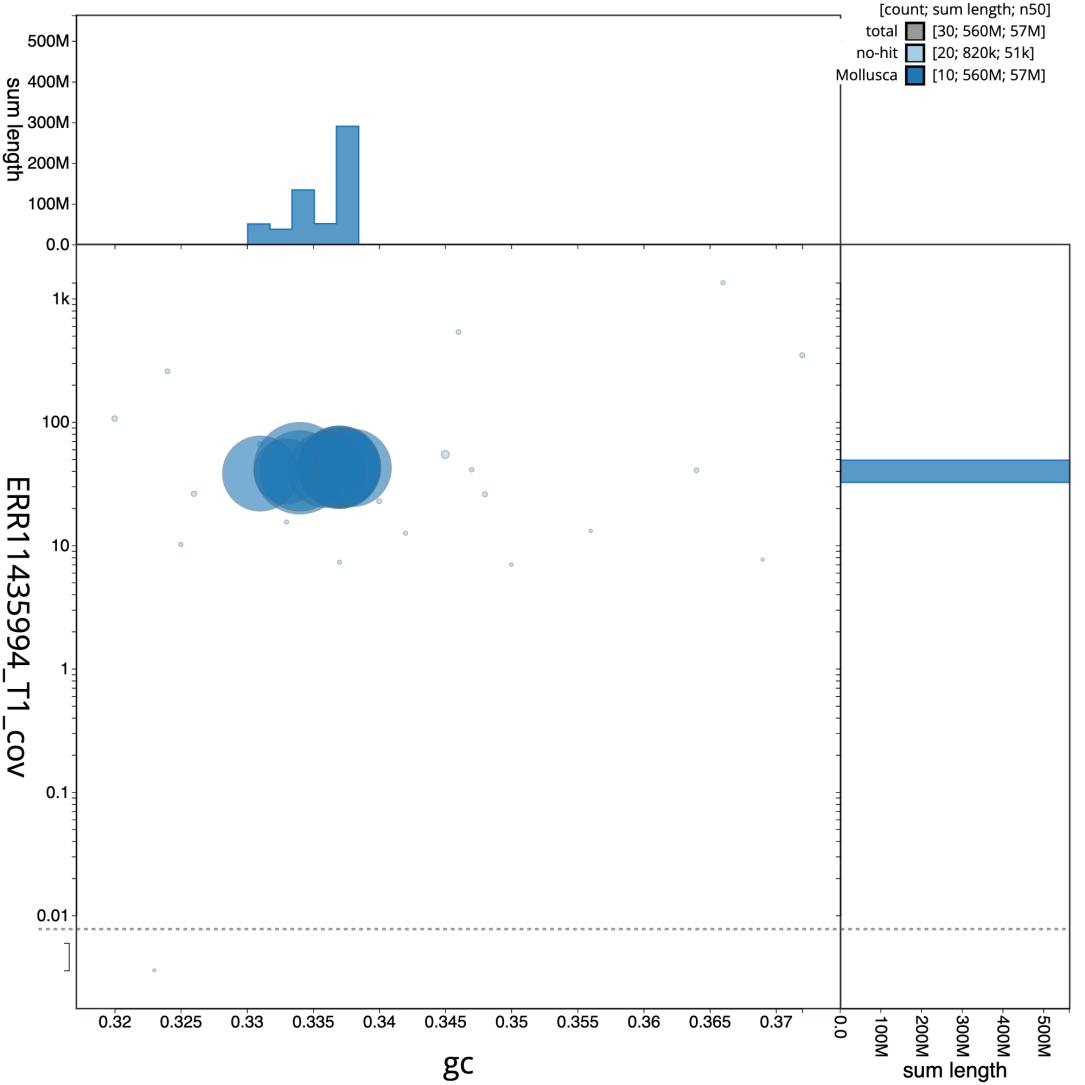
Genome assembly of *Magallana gigas*, xbMagGiga1.1: BlobToolKit GC-coverage plot. Sequences are coloured by phylum. Circles are sized in proportion to sequence length. Histograms show the distribution of sequence length sum along each axis. An interactive version of this figure is available at https://blobtoolkit.genomehubs.org/view/Magallana_gigas/dataset/GCA_963853765.1/blob.

**Figure 4.**
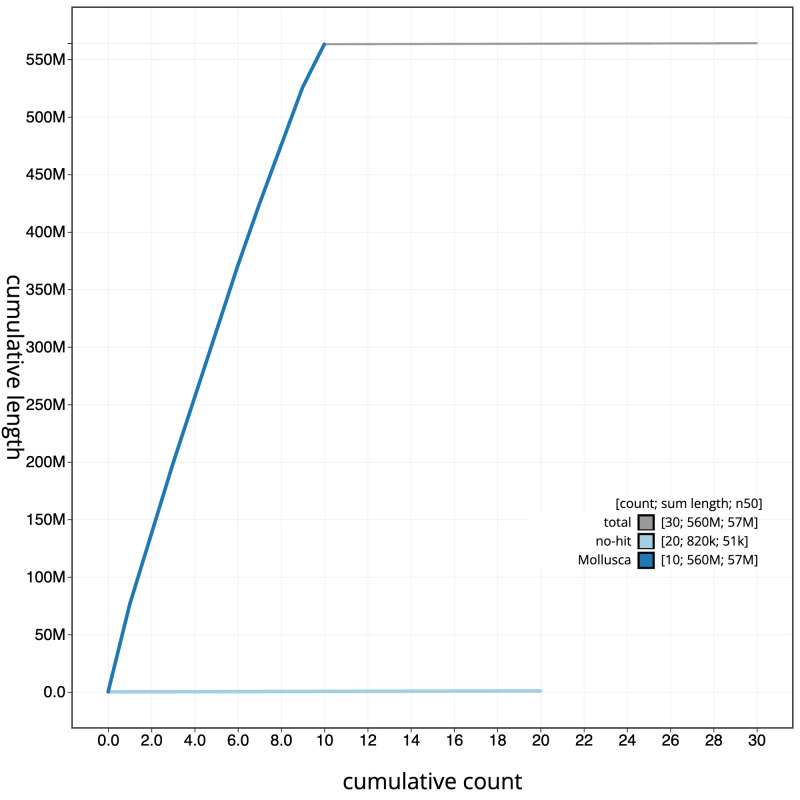
Genome assembly of *Magallana gigas*, xbMagGiga1.1: BlobToolKit cumulative sequence plot. The grey line shows cumulative length for all sequences. Coloured lines show cumulative lengths of sequences assigned to each phylum using the buscogenes taxrule. An interactive version of this figure is available at https://blobtoolkit.genomehubs.org/view/Magallana_gigas/dataset/GCA_963853765.1/cumulative.

**Figure 5.**
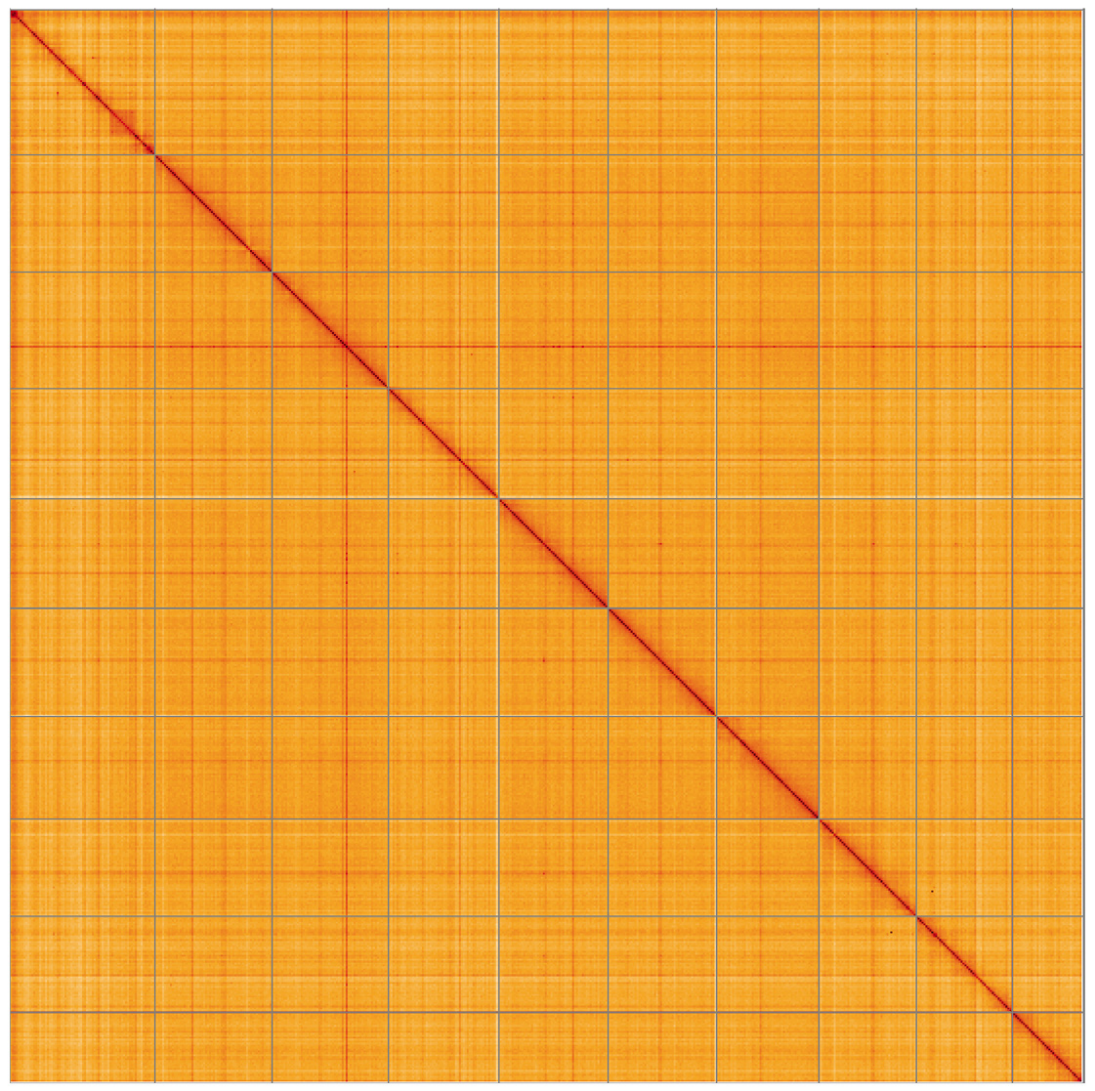
Genome assembly of *Magallana gigas*, xbMagGiga1.1: Hi-C contact map of the xbMagGiga1.1 assembly, visualised using HiGlass. Chromosomes are shown in order of size from left to right and top to bottom. An interactive version of this figure may be viewed at https://genome-note-higlass.tol.sanger.ac.uk/l/?d=BM-Ctut_Q3GGlAPdqRWtLQ.

The final assembly has a total length of 564.0 Mb in 29 sequence scaffolds with a scaffold N50 of 57.3 Mb ( Table 1). The snail plot in Figure 2 provides a summary of the assembly statistics, while the distribution of assembly scaffolds on GC proportion and coverage is shown in Figure 3. The cumulative assembly plot in Figure 4 shows curves for subsets of scaffolds assigned to different phyla. Most (99.85%) of the assembly sequence was assigned to 10 chromosomal-level scaffolds. Chromosome-scale scaffolds confirmed by the Hi-C data are named in order of size ( Figure 5; Table 2). While not fully phased, the assembly deposited is of one haplotype. Contigs corresponding to the second haplotype have also been deposited. The mitochondrial genome was also assembled and can be found as a contig within the multifasta file of the genome submission.

**Table 1.** Genome data for *Magallana gigas*, xbMagGiga1.1.

Project accession data		
Assembly identifier	xbMagGiga1.1	
Species	*Magallana gigas*	
Specimen	xbMagGiga1	
NCBI taxonomy ID	2171618	
BioProject	PRJEB61921	
BioSample ID	SAMEA7536694	
Isolate information	xbMagGiga1, muscle (DNA, Hi-C and RNA sequencing)	
Assembly metrics *		*Benchmark*
Consensus quality (QV)	59.7	*≥ 50*
*k*-mer completeness	97.62% (combined assemblies)	*≥ 95%*
BUSCO **	C:96.5%[S:96.2%,D:0.3%], F:0.3%,M:3.2%,n:5,295	*C ≥ 95%*
Percentage of assembly mapped to chromosomes	99.85%	*≥ 95%*
Sex chromosomes	None	*localised homologous pairs*
Organelles	Mitochondrial genome: 18.23 kb	*complete single alleles*
Raw data accessions		
PacificBiosciences Sequel IIe, Sequel II	ERR11435993, ERR11435994	
Hi-C Illumina	ERR11439652	
PolyA RNA-Seq Illumina	ERR11439653	
Genome assembly		
Assembly accession	GCA_963853765.1	
*Accession of alternate haplotype*	GCA_963853805.1	
Span (Mb)	564.0	
Number of contigs	185	
Contig N50 length (Mb)	7.3	
Number of scaffolds	29	
Scaffold N50 length (Mb)	57.3	
Longest scaffold (Mb)	76.07	
Genome annotation of assembly GCA_963853765.1 at Ensembl		
Number of protein-coding genes	19,775		
Number of non-coding genes	6,806		
Number of gene transcripts	37,189		

* Assembly metric benchmarks are adapted from column VGP-2020 of “Table 1: Proposed standards and metrics for defining genome assembly quality” from Rhie *et al.* (2021). ** BUSCO scores based on the mollusca_odb10 BUSCO set using version v5.4.3. C = complete [S = single copy, D = duplicated], F = fragmented, M = missing, n = number of orthologues in comparison. A full set of BUSCO scores is available at https://blobtoolkit.genomehubs.org/view/Magallana_gigas/dataset/GCA_963853765.1/busco.

**Table 2.** Chromosomal pseudomolecules in the genome assembly of *Magallana gigas*, xbMagGiga1.

INSDC accession	Chromosome	Length (Mb)	GC%
OY970745.1	1	76.07	33.5
OY970746.1	2	61.47	33.5
OY970747.1	3	61.04	33.5
OY970748.1	4	57.95	33.5
OY970749.1	5	57.27	33.5
OY970750.1	6	56.91	33.5
OY970751.1	7	53.67	34.0
OY970752.1	8	51.13	33.5
OY970753.1	9	50.36	33.0
OY970754.1	10	37.31	33.5
OY970755.1	MT	0.02	36.5

## Genome annotation report

The *Magallana gigas* genome assembly (GCA_963853765.1) was annotated at the European Bioinformatics Institute (EBI) on Ensembl Rapid Release. The resulting annotation includes 37,189 transcribed mRNAs from 19,775 protein-coding and 6,806 non-coding genes ( Table 2; https://rapid.ensembl.org/Magallana_gigas_GCA_963853765.1/Info/Index). The average transcript length is 9,924.07. There are 1.40 coding transcripts per gene and 7.57 exons per transcript.

The estimated Quality Value (QV) of the final assembly is 59.7 with *k*-mer completeness of 97.62%, and the assembly has a BUSCO v5.4.2 completeness of 96.5% (single = 96.2%, duplicated = 0.3%), using the mollusca_odb10 reference set ( *n* = 5,295).

Metadata for specimens, BOLD barcode results, spectra estimates, sequencing runs, contaminants and pre-curation assembly statistics are given at https://links.tol.sanger.ac.uk/species/2171618.

## Methods

### Sample acquisition and nucleic acid extraction

A *Magallana gigas* (specimen ID MBA-200724-001A, ToLID xbMagGiga1) was collected from Noss Mayo, Devon, UK (latitude 50.31, longitude –4.05) on 2020-07-24. The specimen was collected by hand and placed in a sample bag. The specimen was collected and identified by Rob Mrowicki (Marine Biological Association) and preserved in liquid nitrogen.

The workflow for high molecular weight (HMW) DNA extraction at the Wellcome Sanger Institute (WSI) Tree of Life Core Laboratory includes a sequence of core procedures: sample preparation; sample homogenisation, DNA extraction, fragmentation, and clean-up. In sample preparation, the xbMagGiga1 sample was weighed and dissected on dry ice ( Jay *et al.*, 2023). For sample homogenisation, muscle tissue was cryogenically disrupted using the Covaris cryoPREP ^®^ Automated Dry Pulverizer ( Narváez-Gómez *et al.*, 2023).

HMW DNA was extracted using the Manual MagAttract v1 protocol ( Strickland *et al.*, 2023b). DNA was sheared into an average fragment size of 12–20 kb in a Megaruptor 3 system with speed setting 30 ( Todorovic *et al.*, 2023). Sheared DNA was purified by solid-phase reversible immobilisation ( Strickland *et al.*, 2023a): in brief, the method employs a 1.8X ratio of AMPure PB beads to sample to eliminate shorter fragments and concentrate the DNA. The concentration of the sheared and purified DNA was assessed using a Nanodrop spectrophotometer and Qubit Fluorometer and Qubit dsDNA High Sensitivity Assay kit. Fragment size distribution was evaluated by running the sample on the FemtoPulse system.

RNA was extracted from muscle tissue of xbMagGiga1 in the Tree of Life Laboratory at the WSI using the RNA Extraction: Automated MagMax™ *mir*Vana protocol ( do Amaral *et al.*, 2023). The RNA concentration was assessed using a Nanodrop spectrophotometer and a Qubit Fluorometer using the Qubit RNA Broad-Range Assay kit. Analysis of the integrity of the RNA was done using the Agilent RNA 6000 Pico Kit and Eukaryotic Total RNA assay.

Protocols developed by the WSI Tree of Life laboratory are publicly available on protocols.io ( Denton *et al.*, 2023).

### Sequencing

Pacific Biosciences HiFi circular consensus DNA sequencing libraries were constructed according to the manufacturers’ instructions. Poly(A) RNA-Seq libraries were constructed using the NEB Ultra II RNA Library Prep kit. DNA and RNA sequencing was performed by the Scientific Operations core at the WSI on Pacific Biosciences Sequel II and Sequel IIe (HiFi) and Illumina HiSeq 4000 (RNA-Seq) instruments. Hi-C data were also generated from muscle tissue of xbMagGiga1 using the Arima2 kit and sequenced on the Illumina NovaSeq 6000 instrument.

### Genome assembly and curation

Assembly was carried out with Hifiasm ( Cheng *et al.*, 2021) and haplotypic duplication was identified and removed with purge_dups ( Guan *et al.*, 2020). The assembly was then scaffolded with Hi-C data ( Rao *et al.*, 2014) using YaHS ( Zhou *et al.*, 2023). The assembly was checked for contamination and corrected using the TreeVal pipeline ( Pointon *et al.*, 2023). Manual curation was performed using JBrowse2 ( Diesh *et al.*, 2023), HiGlass ( Kerpedjiev *et al.*, 2018) and PretextView ( Harry, 2022). The mitochondrial genome was assembled using MitoHiFi ( Uliano-Silva *et al.*, 2023), which runs MitoFinder ( Allio *et al.*, 2020) or MITOS ( Bernt *et al.*, 2013) and uses these annotations to select the final mitochondrial contig and to ensure the general quality of the sequence.

### Genome annotation method

The Ensembl Genebuild annotation system ( Aken *et al*., 2016) was used to generate annotation for the *Magallana gigas* assembly (GCA_963853765.1) in Ensembl Rapid Release at the EBI. Annotation was created primarily through alignment of transcriptomic data to the genome, with gap filling via protein-to-genome alignments of a select set of proteins from UniProt ( UniProt Consortium, 2019).

### Final assembly evaluation

The final assembly was post-processed and evaluated with the three Nextflow ( Di Tommaso *et al.*, 2017) DSL2 pipelines “sanger-tol/readmapping” ( Surana *et al.*, 2023a), “sanger-tol/genomenote” ( Surana *et al.*, 2023b), and “sanger-tol/blobtoolkit” ( Muffato *et al.*, 2024). The pipeline sanger-tol/readmapping aligns the Hi-C reads with bwa-mem2 ( Vasimuddin *et al.*, 2019) and combines the alignment files with SAMtools ( Danecek *et al.*, 2021). The sanger-tol/genomenote pipeline transforms the Hi-C alignments into a contact map with BEDTools ( Quinlan & Hall, 2010) and the Cooler tool suite ( Abdennur & Mirny, 2020), which is then visualised with HiGlass ( Kerpedjiev *et al.*, 2018). It also provides statistics about the assembly with the NCBI datasets ( Sayers *et al.*, 2024) report, computes *k*-mer completeness and QV consensus quality values with FastK and MerquryFK, and a completeness assessment with BUSCO ( Manni *et al.*, 2021).

The sanger-tol/blobtoolkit pipeline is a Nextflow port of the previous Snakemake Blobtoolkit pipeline ( Challis *et al.*, 2020). It aligns the PacBio reads with SAMtools and minimap2 ( Li, 2018) and generates coverage tracks for regions of fixed size. In parallel, it queries the GoaT database ( Challis *et al.*, 2023) to identify all matching BUSCO lineages to run BUSCO ( Manni *et al.*, 2021). For the three domain-level BUSCO lineage, the pipeline aligns the BUSCO genes to the Uniprot Reference Proteomes database ( Bateman *et al.*, 2023) with DIAMOND ( Buchfink *et al.*, 2021) blastp. The genome is also split into chunks according to the density of the BUSCO genes from the closest taxonomically lineage, and each chunk is aligned to the Uniprot Reference Proteomes database with DIAMOND blastx. Genome sequences that have no hit are then chunked with seqtk and aligned to the NT database with blastn ( Altschul *et al.*, 1990). All those outputs are combined with the blobtools suite into a blobdir for visualisation.

All three pipelines were developed using the nf-core tooling ( Ewels *et al.*, 2020), use MultiQC ( Ewels *et al.*, 2016), and make extensive use of the Conda package manager, the Bioconda initiative ( Grüning *et al.*, 2018), the Biocontainers infrastructure ( da Veiga Leprevost *et al.*, 2017), and the Docker ( Merkel, 2014) and Singularity ( Kurtzer *et al.*, 2017) containerisation solutions.

Table 3 contains a list of relevant software tool versions and sources.

**Table 3.** Software tools: versions and sources.

Software tool	Version	Source
BEDTools	2.30.0	https://github.com/arq5x/bedtools2
Blast	2.14.0	ftp://ftp.ncbi.nlm.nih.gov/blast/executables/blast+/
BlobToolKit	4.3.7	https://github.com/blobtoolkit/blobtoolkit
BUSCO	5.4.3	https://gitlab.com/ezlab/busco
BUSCO	5.4.3 and 5.5.0	https://gitlab.com/ezlab/busco
bwa-mem2	2.2.1	https://github.com/bwa-mem2/bwa-mem2
Cooler	0.8.11	https://github.com/open2c/cooler
DIAMOND	2.1.8	https://github.com/bbuchfink/diamond
fasta_windows	0.2.4	https://github.com/tolkit/fasta_windows
FastK	427104ea91c78c3b8b8b49f1a7d6bbeaa869ba1c	https://github.com/thegenemyers/FASTK
GoaT CLI	0.2.5	https://github.com/genomehubs/goat-cli
Hifiasm	0.16.1-r375	https://github.com/chhylp123/hifiasm
HiGlass	1.11.6	https://github.com/higlass/higlass
HiGlass	44086069ee7d4d3f6f3f0012569789ec138f42b84 aa44357826c0b6753eb28de	https://github.com/higlass/higlass
MerquryFK	d00d98157618f4e8d1a9190026b19b471055b22e	https://github.com/thegenemyers/MERQURY.FK
MitoHiFi	3	https://github.com/marcelauliano/MitoHiFi
MultiQC	1.14, 1.17, and 1.18	https://github.com/MultiQC/MultiQC
NCBI Datasets	15.12.0	https://github.com/ncbi/datasets
Nextflow	23.04.0-5857	https://github.com/nextflow-io/nextflow
PretextView	0.2	https://github.com/wtsi-hpag/PretextView
purge_dups	1.2.5	https://github.com/dfguan/purge_dups
samtools	1.16.1, 1.17, and 1.18	https://github.com/samtools/samtools
sanger-tol/genomenote	1.1.1	https://github.com/sanger-tol/genomenote
sanger-tol/readmapping	1.2.1	https://github.com/sanger-tol/readmapping
Seqtk	1.3	https://github.com/lh3/seqtk
Singularity	3.9.0	https://github.com/sylabs/singularity
TreeVal	1.0.0	https://github.com/sanger-tol/treeval
YaHS	1.2a.2	https://github.com/c-zhou/yahs

### Wellcome Sanger Institute – Legal and Governance

The materials that have contributed to this genome note have been supplied by a Darwin Tree of Life Partner. The submission of materials by a Darwin Tree of Life Partner is subject to the **‘Darwin Tree of Life Project Sampling Code of Practice’**, which can be found in full on the Darwin Tree of Life website here. By agreeing with and signing up to the Sampling Code of Practice, the Darwin Tree of Life Partner agrees they will meet the legal and ethical requirements and standards set out within this document in respect of all samples acquired for, and supplied to, the Darwin Tree of Life Project. 

Further, the Wellcome Sanger Institute employs a process whereby due diligence is carried out proportionate to the nature of the materials themselves, and the circumstances under which they have been/are to be collected and provided for use. The purpose of this is to address and mitigate any potential legal and/or ethical implications of receipt and use of the materials as part of the research project, and to ensure that in doing so we align with best practice wherever possible. The overarching areas of consideration are:

• Ethical review of provenance and sourcing of the material

• Legality of collection, transfer and use (national and international) 

Each transfer of samples is further undertaken according to a Research Collaboration Agreement or Material Transfer Agreement entered into by the Darwin Tree of Life Partner, Genome Research Limited (operating as the Wellcome Sanger Institute), and in some circumstances other Darwin Tree of Life collaborators.

## Data Availability

European Nucleotide Archive: *Magallana gigas* (Pacific oyster). Accession number PRJEB61921; https://identifiers.org/ena.embl/PRJEB61921 ( Wellcome Sanger Institute, 2023). The genome sequence is released openly for reuse. The *Magallana gigas* genome sequencing initiative is part of the Darwin Tree of Life (DToL) project. All raw sequence data and the assembly have been deposited in INSDC databases. Raw data and assembly accession identifiers are reported in Table 1.

## Author information

Members of the Marine Biological Association Genome Acquisition Lab are listed here: https://doi.org/10.5281/zenodo.8382513.

Members of the Darwin Tree of Life Barcoding collective are listed here: https://doi.org/10.5281/zenodo.4893703.

Members of the Wellcome Sanger Institute Tree of Life Management, Samples and Laboratory team are listed here: https://doi.org/10.5281/zenodo.10066175.

Members of Wellcome Sanger Institute Scientific Operations: Sequencing Operations are listed here: https://doi.org/10.5281/zenodo.10043364.

Members of the Wellcome Sanger Institute Tree of Life Core Informatics team are listed here: https://doi.org/10.5281/zenodo.10066637.

Members of the Tree of Life Core Informatics collective are listed here: https://doi.org/10.5281/zenodo.5013541.

Members of the Darwin Tree of Life Consortium are listed here: https://doi.org/10.5281/zenodo.4783558.
