# Supplementary material for: The genome sequence of the Pacific oyster, Magallana gigas (Thunberg, 1793)
Source: Wellcome Open Res. 2025 Feb 3;9:284. Originally published 2024 May 21. [Version 2] doi: 10.12688/wellcomeopenres.22255.2 (PMC11267148; doi:10.12688/wellcomeopenres.22255.2)
Supplement: Peer review history

# Reviewer report: Reviewer response for version 2

**DOI:** 10.21956/wellcomeopenres.26051.r118092

**Published:** 2025-02-11

**Authors:** Qinggang Xue

**Affiliations:** 1 Zhejiang Wanli University, Ningbo, Zhejiang, China

The authors have addressed my concerns in the revision.

Are sufficient details of methods and materials provided to allow replication by others?

Partly

Is the rationale for creating the dataset(s) clearly described?

Partly

Are the datasets clearly presented in a useable and accessible format?

Yes

Are the protocols appropriate and is the work technically sound?

Yes

Reviewer Expertise:

Mollusk physiology and immunity, molecular and functional evolution of immune and stress response related proteins in mollusks

I confirm that I have read this submission and believe that I have an appropriate level of expertise to confirm that it is of an acceptable scientific standard.

# Reviewer report: Reviewer response for version 2

**DOI:** 10.21956/wellcomeopenres.26051.r118093

**Published:** 2025-02-05

**Authors:** Bassem Allam

**Affiliations:** 1 Stony Brook University, Stony Brook, New York, USA

Good work, but the shortcomings identified by all three reviewers have not been addressed (rationale for another assembly of the species and basic benchmarking of the current assembly with previous chromosome-level assemblies).

Are sufficient details of methods and materials provided to allow replication by others?

Yes

Is the rationale for creating the dataset(s) clearly described?

Partly

Are the datasets clearly presented in a useable and accessible format?

Yes

Are the protocols appropriate and is the work technically sound?

Yes

Reviewer Expertise:

Host-pathogen interactions, Shellfish genomics, Shellfish aquaculture

I confirm that I have read this submission and believe that I have an appropriate level of expertise to confirm that it is of an acceptable scientific standard.

# Reviewer report: Reviewer response for version 1

**DOI:** 10.21956/wellcomeopenres.24532.r88482

**Published:** 2024-08-08

**Authors:** Qinggang Xue

**Affiliations:** 1 Zhejiang Wanli University, Ningbo, Zhejiang, China

This is a data note for the genome sequence of a Pacific oyster individual collected in UK. Based on the reported assessment parameters, it was a high-quality genome assembly. However, there are a few points that should be further addressed or clarified.

1. There are already 2 high quality chromosome-level genome sequence assemblies in the public sequence databases, and one of them was done in the UK. What were the rationales for the present assembly? 
2. It should be made more direct and clearer that the note represented the third complete genome assembly of a same oyster species. This can help to avoid potential confusions in the research community as a different scientific name was used in this work.  It should also be addressed that renaming *Crassostrea gigas* as *Magallana gigas* is not yet a scientific consensus in the oyster research community.
3. Because of the existence 2 previous assemblies, readers may expect some brief comparison between this new completed one and the previous two.
4. The authors detailed the pipelines for assembly evaluation, but did not describe how manual assembly curation was done. The latter is an important piece of information.
5. Where is the RNA-seq data?

Are sufficient details of methods and materials provided to allow replication by others?

Partly

Is the rationale for creating the dataset(s) clearly described?

Partly

Are the datasets clearly presented in a useable and accessible format?

Yes

Are the protocols appropriate and is the work technically sound?

Yes

Reviewer Expertise:

Mollusk physiology and immunity, molecular and functional evolution of immune and stress response related proteins in mollusks

I confirm that I have read this submission and believe that I have an appropriate level of expertise to confirm that it is of an acceptable scientific standard, however I have significant reservations, as outlined above.

# Reviewer report: Reviewer response for version 1

**DOI:** 10.21956/wellcomeopenres.24532.r88485

**Published:** 2024-07-23

**Authors:** Jian-Wen Qiu

**Affiliations:** 1 Department of Biology, Hong Kong Baptist University, Hong Kong, Hong Kong

This project aims to produce a high-quality genome sequence for the Pacific oyster. The methods used were state-of-the-art and well-described, and the assembly is of high quality, as shown by the completeness and continuity statistics. I have only two minor comments about the research background:

 1. The distribution range is Northern Asia, not Northern and Southern Asia. Along the coasts of Southern Asia, there are other native oyster species such as the Hong Kong oyster and the "Portuguese" oyster.

 2. In addition to Qi et al. (2021) cited by the authors, there are at least two versions of the Pacific genome available i.e,

- Zhang G, Fang X, Guo X, Li L, et al.: The oyster genome reveals stress adaptation and complexity of shell formation. *Nature*. 2012; **490** (7418): 49-54;
- Peñaloza C, Gutierrez AP, Eöry L, Wang S, et al.: A chromosome-level genome assembly for the Pacific oyster Crassostrea gigas. *Gigascience*. 2021; **10** (3).

The authors may want to state the necessity to produce this version of the genome.

Are sufficient details of methods and materials provided to allow replication by others?

Yes

Is the rationale for creating the dataset(s) clearly described?

Partly

Are the datasets clearly presented in a useable and accessible format?

Yes

Are the protocols appropriate and is the work technically sound?

Yes

Reviewer Expertise:

genomics of marine invertebrates, especially molluscs and annelids.

I confirm that I have read this submission and believe that I have an appropriate level of expertise to confirm that it is of an acceptable scientific standard.

## References

[1] : The oyster genome reveals stress adaptation and complexity of shell formation. *Nature* .2012;490(7418) : 10.1038/nature11413 49-54 10.1038/nature11413 22992520

[2] : A chromosome-level genome assembly for the Pacific oyster Crassostrea gigas. *Gigascience* .2021;10(3) : 10.1093/gigascience/giab020 10.1093/gigascience/giab020 33764468 PMC7992393

# Reviewer report: Reviewer response for version 1

**DOI:** 10.21956/wellcomeopenres.24532.r86382

**Published:** 2024-07-17

**Authors:** Bassem Allam

**Affiliations:** 1 Stony Brook University, Stony Brook, New York, USA

This is a straightforward and nicely written paper. The authors provide rationale supporting the economic and ecological importance of the target species and its broad distribution worldwide. The techniques and approaches used are robust and state-of-the-art. The quality of the graphics is excellent, and the tables provide well-structured information.

 The only shortcoming identified pertains to the lack of information about the availability of previous genome assemblies. This is shortly mentioned at the end of the background section but more information is needed to inform the reader about the limitations of current assemblies, if any. In other words, why do we need another assembly of the M. gigas genome? More importantly, the paper lacks a comparative assessment of the current assembly with previous assemblies. Such a comparison would further highlight the importance of the current work. The authors indicate that “a complete genome sequence will thereby provide further information to….”, which implies that the previous assemblies may not have been necessarily “complete”. Such a statement needs supporting information.

**Minor editorial imperfections:**

- There is an extra “v” between “BUSCO” and “completeness” under “Genome sequencing report”.
- There is extraneous text (authors’ comments) in the capture of Figure 1.
- The online version of Figure 4 at https://blobtoolkit.genomehubs.org/view/Magallana_gigas/dataset/GCA_963853765.1/cumulative is not really interactive (at least on my screen), so dubbing it as such in the figure capture is not accurate.

Are sufficient details of methods and materials provided to allow replication by others?

Yes

Is the rationale for creating the dataset(s) clearly described?

Partly

Are the datasets clearly presented in a useable and accessible format?

Yes

Are the protocols appropriate and is the work technically sound?

Yes

Reviewer Expertise:

Host-pathogen interactions, Shellfish genomics, Shellfish aquaculture

I confirm that I have read this submission and believe that I have an appropriate level of expertise to confirm that it is of an acceptable scientific standard.
